# Supersaccharated Milk in Adult Dyspepsias with Gastralgic Crises and Vomiting

**Published:** 1916-05

**Authors:** 


**Supersaccharated Milk in Adult Dyspepsias with Gastralgic Crises and Vomiting.** G. Variot and O. Burileano, Soc. Med. des Hop. Nov. 5, 1915, advocates the addition of 10% of saccharose to milk, giving 2 liters a day to women, 3 to men. Apparently, reference is made to hyperchlorhydric cases. The urine does not show sugar.

